# The Influence of Body Mass Index on Monocytes and Eosinophil Levels and Their Relationship With Spirometric Parameters in Children and Adolescents With Bronchial Asthma

**DOI:** 10.1155/carj/7534325

**Published:** 2025-05-12

**Authors:** Regina N. Khramova, Tatyana I. Eliseeva, Dmitry Y. Ovsyannikov, Elena V. Tush, Maxim A. Karpenko, Anastasia A. Shamrikova, Nailya I. Kubysheva, Vilya A. Bulgakova, Olga V. Khaletskaya, Natalia A. Geppe, Ildar Z. Batyrshin

**Affiliations:** ^1^Department of Hospital Pediatrics, Privolzhsky Research Medical University, Nizhny Novgorod 603005, Russia; ^2^Department of Pediatrics, Medical Institute, Peoples' Friendship University of Russia (RUDN University), 6 Miklukho-Maklaya St., Moscow 117198, Russia; ^3^Faculty of Informatics, Mathematics and Computer Science, Direction of Business Informatics, National Research University Higher School of Economics, Nizhny Novgorod 603014, Russia; ^4^Kazan Federal University, Kremlyovskaya St, 18, Kazan 420000, Russia; ^5^Pediatrics and Child Health Research Institute in Petrovsky National Research Centre of Surgery, Moscow 119991, Russia; ^6^Pirogov Russian National Research Medical University, Moscow 117997, Russia; ^7^Sechenov First Moscow State Medical University, Moscow 119992, Russia; ^8^Instituto Politécnico Nacional, Centro de Investigación en Computación (CIC-IPN), Av. Juan de Dios Bátiz S/N, Nueva Industrial Vallejo, Gustavo A. Madero, Ciudad de México 07738, Mexico

**Keywords:** adolescent, asthma bronchial, body mass index, child, eosinophils, monocytes, spirometry

## Abstract

**Objectives:** Excess adipose tissue induces low-intensity inflammation, which is an important pathogenetic factor adversely affecting the course of bronchial asthma (BA) in overweight/obese patients. The key effector cells of this inflammation are monocytes and macrophages. However, there are currently no studies characterising the effect of body mass index (BMI) on peripheral blood monocyte levels in children and adolescents with BA and their relationship with spirometric parameters reflecting bronchial patency. The aim of this study was to investigate the effect of BMI on peripheral blood monocyte and eosinophil levels and their relationship with spirometric parameters in children and adolescents with asthma.

**Methods:** A single-centre, observational cross-sectional study was conducted. A total of 212 patients with asthma aged 7–17 years were studied. Anthropometric and spirometric parameters and the cellular composition of peripheral blood were evaluated. The children were divided into two groups: Group 1: with normal body weight (BW) and Group 2: with overweight/obesity.

**Results:** In overweight/obesity patients, the number of peripheral blood monocytes (0.62 ± 0.19) was significantly higher compared to the group of normal weight patients (0.54 ± 0.15, *p* < 0.001). In contrast, eosinophil levels were statistically lower in the overweight/obesity group (0.22 [0.12; 0.42]) than in the normal weight patients (0.30 [0.14; 0.56], *p* = 0.039). A statistically significant negative correlation was found between the absolute number of monocytes and z FEV1/FVC, z MMEF_25-75_ in the overweight/obesity group (*R* = −0.32, *p* = 0.005, *R* = −0.30, *p* = 0.007, respectively) and a statistically significant negative correlation between eosinophil count and z FEV1/FVC, z MMEF25-75 in normal weight patients (*R* = −0.20, *p* = 0.021, *R* = −0.22, *p* = 0.010, respectively).

**Conclusions:** The results obtained may indicate a modifying effect of overweight/obesity on inflammation endotypes in children and adolescents with BA.

## 1. Introduction

Bronchial asthma (BA) is a common chronic inflammatory disease of the airway characterised by variable obstruction and airway hyper-responsiveness (AHR) [1–3]. The inflammatory features of BA are largely determined by disease phenotype [4]. In children and adolescents, the most common allergic phenotype of BA is mainly characterised by eosinophilic inflammation. Accordingly, peripheral blood eosinophils are included in the list of its systemic markers. A negative relationship between eosinophil count and spirometric indicators, including the FEV1/FVC ratio, has been demonstrated [5]. Another clinically significant phenotype of BA is that associated with overweight (OW) and obesity (OB). OW and OB are known to be factors that adversely modify asthma and worsen its course [6, 7].

The mechanisms underlying the adverse effects of OW and OB on the course of BA are not fully determined. However, it is known that OB is characterised by an increase in the size and number of adipocytes in the adipose tissue and is accompanied by their excessive synthesis of pro-inflammatory cytokines. These cytokines contribute to the activation of circulating monocytes and adipose tissue macrophages [8–10]. Recent publications indicate that the altered inflammation in BA patients in combination with OW/OB also involves activation of other factors associated with monocytes [11, 12].

Monocytes and macrophages are thus key cells that characterise the low-intensity systemic inflammation induced by excess adipose tissue [13–16]. Literature data show that OB is characterised by a higher monocytes number in the peripheral blood compared to normal body weight [17]. Researchers are currently investigating the involvement of monocytes in the pathogenesis of asthma, including fatal asthma [18–21]. Furthermore, OB-induced systemic inflammation has been shown to be associated with monocyte dysfunction in BA patients [22, 23]. However, we did not find any studies on peripheral blood monocytes count in children and adolescents with BA and different body mass indexes (BMI). There is also currently no information on the influence of BMI on the relationship between blood monocytes count and spirometric parameters in asthmatics. We found only one study showing an inverse correlation between total peripheral blood monocytes count and FEV1/FVC in severe asthmatics [24]. However, this study did not analyse the effect of BMI on this relationship.

The aim of this study was to investigate the effect of BMI on peripheral blood monocyte and eosinophil levels and their relationship with spirometric parameters in children and adolescents with asthma.

## 2. Materials and Methods

### 2.1. Research Design

A single-centre observational cross-sectional study was conducted.

### 2.2. Study Conditions

The study was conducted at the Children's City Clinical Hospital No. 1 in Nizhny Novgorod, Russia, in 2017–2023.

### 2.3. Study Participants

The study included 212 patients with atopic asthma aged 7–17 years who were being treated for the disease. Family history associated with atopy (asthma, allergic rhinitis, conjunctivitis, atopic dermatitis and urticaria) was assessed, and sensitisation to the main aeroallergens (house dust mite, cat, dog, pollen allergens) was assessed in vivo (prick test) or in vitro (with determination of specific IgE).

### 2.4. Inclusion Criteria

1. Diagnosis of asthma according to the current international consensus documents (GINA, 2016–2021),
2. Age of the patients between 7 and 17 years,
3. Oxygen saturation ≥ 96% or SpO_2_ ≥ 96%.

### 2.5. Exclusion Criteria

1. Patients with a BMI of less than-1Z (274 patients were initially enrolled, of whom 62 had a BMI less than -1Z),
2. The presence of acute infectious diseases and fever,
3. The presence of diabetes mellitus, autoimmune diseases, primary immunodeficiency, cancer, atopic dermatitis, parasitic diseases,
4. Severe BA [1],
5. Systemic use of glucocorticoids,
6. Epilepsy drugs, nonsteroidal anti-inflammatory drugs, angiotensin-converting enzyme inhibitors

The study was approved by the Ethics Committee of the Volga Region Research Medical University (Protocol No. 13 dated 10.10.2016). All participants and all primary care providers gave written informed consent.

### 2.6. Data Sources

#### 2.6.1. Anthropometric Indicators

All patients were assessed for basic anthropometric indicators. All measurements were taken without shoes, outer clothing and headgear. Anthropometric parameters (height, body weight and BMI) were estimated using tables developed by the World Health Organization (WHO), taking into account the sex and age of the patients (https://www.who.int/tools/child-growth-standards).

  Calculation of BMI: BMI = body weight (kg)/height (m)^2^.

According to the BMI assessment data in this study, the children were divided into two groups:

  Group 1 (normal weight)—normal body weight, BMI values from -1Z to +1Z,
  Group 2 (overweight [OW]/obesity [OB])—OW and OB, BMI values above +1Z.

### 2.7. Physical Activity of Patients

During the medical history collection, all patients had their physical activity checked before hospitalisation.

All patients were asked about their physical activity before hospitalisation during the medical history.

### 2.8. Spirometry

Spirometric studies were performed using a Masterscreen Pneumospirometer (Jaeger, Germany). The study was performed by certified specialists. The following parameters were evaluated in the analysis of the spirometry data:

  FVC (L) is the forced vital capacity of the lungs, reflecting the volume of the lungs.
  FEV_1_ (L) is forced expiratory volume in 1 s.
  FEV_1_/FVC—the index, which is the main parameter of spirometry for the diagnosis of obstructive disease.
  MMEF_25-75_—maximal mid-expiratory flow between 75% and 25% of the FVC is the average volumetric flow rate during forced expiration in the range of 25%–75% of the forced vital capacity of the lungs.

Spirometry data were measured in absolute values, and the ratio of FEV_1_/FVC ratio was calculated.

In addition, z FVC, z FEV_1_ and z FEV_1_/FVC and z MMEF_25-75_ were calculated using the Global Lung Function Initiative calculator (https://gli-calculator.ersnet.org/index.html), developed with the support of the European Respiratory Society (ERS, https://www.ersnet.org).

### 2.9. Blood Test

All participants underwent peripheral blood examination with counting of erythrocytes, haemoglobin, absolute number of leukocytes, monocytes, eosinophils on an XS series automatic haematological analyser (XS-1000i/XS-800i, SYSMEX CORPORATION, Japan).

The normal values of eosinophils in peripheral blood were estimated to be (0.05–0.45) 10^∗9^/L [25].

Normal values for peripheral blood monocytes were estimated to be (0–0.8) 10^∗9^/L [26].

Determination of serum immunoglobulin E levels was performed using the IgE-ELISA-Best test systems manufactured by Vector-Best JSC (Russia) on the automated enzyme immunoassay analyser ELISA-QS, RADIM GROUP (Italy).

### 2.10. Statistical Analysis

Statistical analysis was performed using Statgraphics Centurion v.16. Quantitative indicators were evaluated for compliance with the normal distribution, the Shapiro–Wilk criterion (with fewer than 50 subjects) or the Kolmogorov–Smirnov criterion (with more than 50 subjects), and indicators of asymmetry and kurtosis were used for this purpose. The data are presented in the form of Me [Q1; Q3], where Me is the median, [Q1; Q3]—1 and 3 quartiles in the case of an abnormal distribution of values and in the form of M ± *σ*, where M is the mean, and *σ* is the standard deviation in the case of their normal distribution. The Mann–Whitney criterion was used to compare quantitative variables in two independent groups. Differences between the two dependent groups were determined using the Wilcoxon W-test. Correlation analysis was performed using Pearson's correlation coefficient for normally distributed variables and Spearman's rank correlation coefficient for abnormally distributed variables.

Categorical data were expressed as absolute values and percentages. Differences were assessed using Pearson's χ^2^ criterion. If the number of expected observations in any of the cells of the four-way table was less than 10, the Fisher exact criterion was used to assess the significance of the differences. Differences were considered statistically significant at *p* < 0.05.

Regression lines were compared using the equation:

(1) $$\begin{array}{c} y=b0+b1^{*}x, \end{array}$$

where *b*0 (slope) is the intersection point of the regression line with the *y*-axis, and b1 (intercept) is the slope of the regression line with the x-axis.

With *p*-values for slope and intercept > 0.05, the regression lines of the compared groups were not statistically significantly different. The regression model was statistically significant if the *p*-value of the model was < 0.05.

This was a pilot study, so the calculation of the sample size was not calculated. The study only included patients who did not have data gaps in the trials that were conducted.

## 3. Results

### 3.1. Study Participants

The study included 212 patients with BA aged 12.0 [9.0; 14.0] years, boys 72.2% (153/212).

Patients in the normal weight and OW/OB groups did not differ statistically significantly in age and gender (Table 1). When comparing anthropometric indicators, patients in the OW/OB group were expected to have statistically significantly higher z weight, z BMI, and z height values than those in the normal body weight group, all *p* < 0.05.

z FEV1/FVC values were statistically significantly lower in the OW/OB group, *p* = 0.006. In the (OW/OB) group, z FVC values were slightly higher than in normal weight patients, the differences were of a trend nature, *p* = 0.080, and z MMEF25-75 values were not different statistically significantly in patients in both groups.

### 3.2. Peripheral Blood Monocytes and Eosinophil Levels in Asthmatic Patients With Different BMIs

The levels of erythrocytes and haemoglobin were slightly higher in the group of patients with OW/OB, the differences were trend (*p* = 0.085 and *p* = 0.099, respectively) (Table 2). The total concentration of IgE was comparable in children with normal body weight and those with OW/OB (*p* = 0.286). The absolute numbers of leukocytes and monocytes in the peripheral blood were statistically significantly higher in the OW and OB group (all *p* < 0.001).

The leukocyte count increased with increasing body weight of the patients and was (6.81 ± 1.60) × 10^9^/L in the normal weight group (*N* = 135), (7.62 ± 1.79) × 10^9^/L in the OW group (*N* = 58) and (7.66 ± 2.01) × 10^9^/L in the OB group (*N* = 19). The differences were statistically significant (*p* = 0.003).

Similar patterns were found when analysing the monocyte level. The absolute values of monocytes were (0.54 ± 0.15) × 10^9^/L in normal weight groups (*N* = 135), (0.60 ± 0.18) × 10^9^/L in OW groups (*N* = 59) and (0.72 ± 0.20) × 10^9^/L in OB groups (*N* = 19). The differences were statistically significant (*p* < 0.001).

The absolute level of eosinophils in peripheral blood was statistically significantly higher in the normal body weight group (*p* = 0.039).

The absolute values of eosinophils in normal weight groups (*N* = 135) were 0.30 [0.14; 0.56], in OW patients (*N* = 58)–0.22 [0.10; 0.42] and in OB patients (*N* = 19)–0.22 [0.14; 0.46], and the differences were not statistically significant (*p* = 0.134).

An excess of monocyte levels above the upper limit of the reference value (0.8 × 10^9^/L) was detected in 8.0% (17/212) of the examined patients. At the same time, monocyte levels were statistically significantly higher in 16.9% (13/77) of OW/OB patients than in 2.9% (4/135) of normal weight asthmatics (*p* = 0.003).

Eosinophil levels exceeding the reference value (0.45 × 10^∗9^/L) were observed in 31.6% (67/212) of the examined patients, in 35.6% (48/135) of the normal body weight subjects and in 24.7% (19/77) of the OW/OB asthmatics. The differences between the groups were not statistically significant (*p* = 0.101).

**Figure 1:**
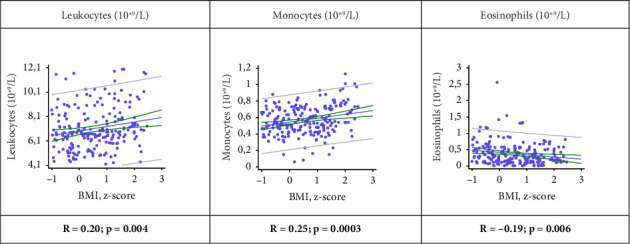
Correlations between leukocytes, monocytes, eosinophil levels, and z BMI. z BMI: z-score BMI (body mass index).

Correlation analysis showed a statistically significant increase in leukocyte and monocyte counts and a decrease in eosinophil levels with increased patient body weight (Figure 1). Statistically significant weak positive correlations were found between absolute leukocyte and monocyte counts and z BMI (*R* = 0.20, *p* = 0.004 and *R* = 0.25, *p* = 0.0003, respectively). There was also a statistically significant weak negative correlation between absolute eosinophil number and z BMI (*R* = −0.19, *p* = 0.006).

**Figure 2:**
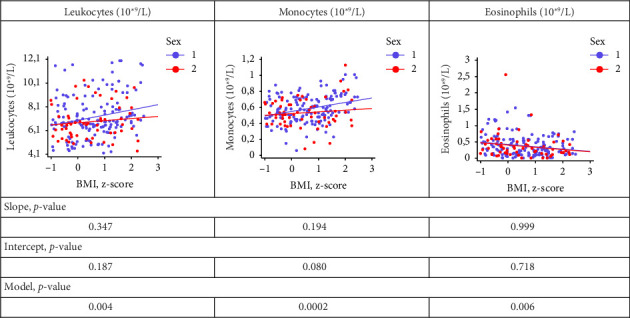
Comparison of regression lines, boys (1) and girls (2): the effect of z BMI on leukocytes, monocytes and eosinophil levels. BMI: body mass index.

We found no differences in the relationships between z BMI and leukocyte, monocyte and eosinophil levels between boys and girls. When the corresponding regression lines were compared between boys and girls, the *p*-values for slope and intercept were greater than 0.05, and the *p*-value for the model was less than 0.05 (Figure 2).

### 3.3. Relationship Between Spirometric Parameters and Peripheral Blood Monocyte and Eosinophil Levels in BA Patients With Different BMIs

**Figure 3:**
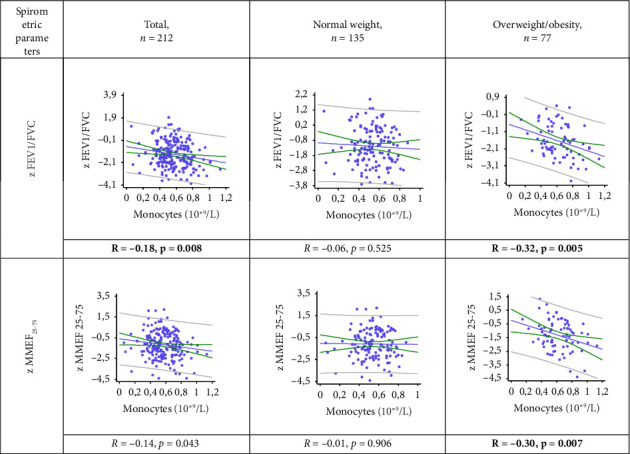
The relationship between spirometric parameters and the absolute number of circulating monocytes in patients with asthma and different BMI: z FVC—z-score forced vital capacity; z FEV_1_/FVC—z-score FEV_1_ (forced expiratory volume in one second)/FVC ratio; z MMEF_25-75_—z-score forced expiratory flow between 25% and 75% of vital capacity.

A statistically significant weak negative correlation was found between absolute monocyte count and z FEV1/FVC in the general and OW/OB groups: *R* = −0.18, *p* = 0.008 and *R* = −0.32, *p* = 0.005, respectively (Figure 3). In the OW/OB group, there was also a statistically significant weak negative correlation between monocyte count and z MMEF25-75 (*R* = −0.30, *p* = 0.007). No relationship was found between the spirometric parameters studied (z FEV1/FVC and z MMEF_25-75_) and monocyte count in normal weight patients (all *p* > 0.05).

**Figure 4:**
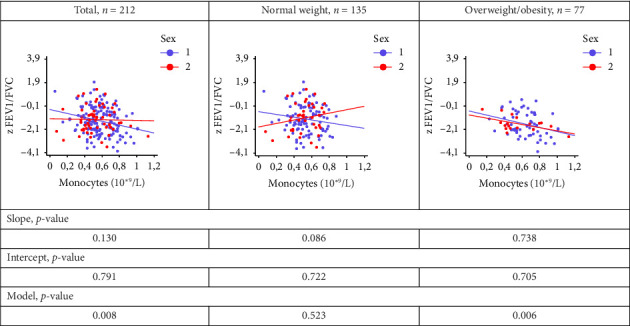
Comparison of regression lines, boys (1) and girls (2): the relationship between monocytes levels and FEV1/FVC in patients with normal body weight and OW/obesity. z FVC—z-score forced vital capacity; z FEV_1_/FVC—z-score FEV_1_ (forced expiratory volume in one second)/FVC ratio.

When comparing the regression lines between boys and girls, reflecting the relationship between the monocyte levels and z FEV1/FVC, the calculated regression models were statistically significant only in the total group and in the OW/OB group (*p* = 0.008, *p* = 0.006, respectively), but not in the normal weight group (*p* = 0.523) (Figure 4). In these groups, the *p* values for the slope and intercept were greater than 0.05 (Figure 4), indicating that there were no differences in these regression lines between the sexes.

**Figure 5:**
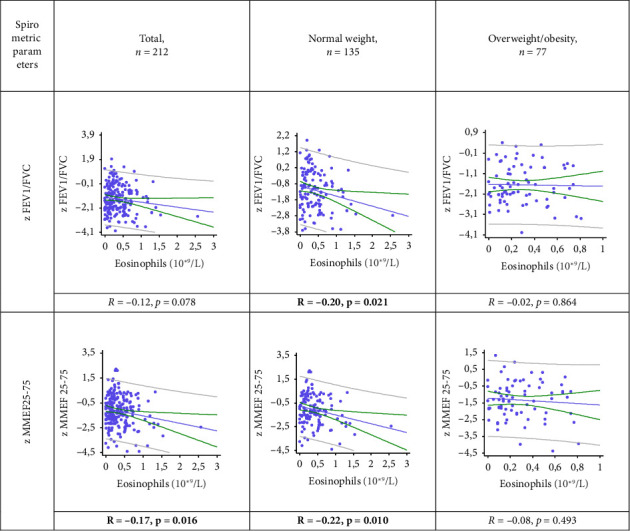
The relationship of spirometric parameters and the absolute number of circulating eosinophils in patients with asthma and different BMI z FVC: z-score forced vital capacity; z FEV1/FVC: z-score FEV1 (forced expiratory volume in one second)/FVC ratio; z MMEF25-75: z-score forced expiratory flow between 25% and 75% of vital capacity.

In normal weight patients, a statistically significant weak negative correlation was found between eosinophil count and z FEV1/FVC and z MMEF_25-75_: *R* = −0.20, *p* = 0.021 and *R* = −0.22, *p* = 0.010, respectively (Figure 5). In the OW/OB group, no relationship was found between these parameters (*p* = 0.864 and *p* = 0.493, respectively).

**Figure 6:**
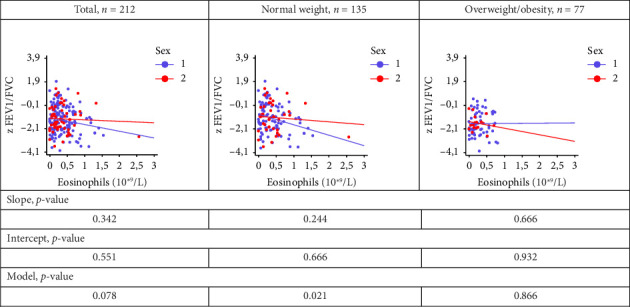
Comparison of regression lines, boys (1) and girls (2): the relationship eosinophil level with FEV_1_/FVC in patients with normal body weight and overweight/obesity. z FVC: z-score forced vital capacity; z FEV_1_/FVC: z-score FEV_1_ (forced expiratory volume in one second)/FVC ratio.

When comparing the regression lines reflecting the relationship between eosinophil level and z FEV1/FVC between boys and girls, the calculated regression models were statistically significant only in the normal weight group (*p* = 0.021), but not in the OW/OB group (*p* = 0.866) (Figure 6). In this group, the *p*-values for slope and intercept were greater than 0.05 (Figure 6), indicating that there were no differences in these regression lines between the sexes.

## 4. Discussion

In this study, we evaluated the effect of OW/OB on the levels of leukocytes, monocytes and eosinophils in peripheral blood, and on the relationship of absolute monocyte and eosinophil counts with spirometric parameters in children and adolescents with asthma.

We showed that leukocyte and monocyte counts were statistically significantly higher, and eosinophil counts were statistically significantly lower in OW/OB patients than in normal weight patients. In addition, we demonstrated a significant inverse correlation between spirometry parameters reflecting bronchial patency (z FEV1/FVC and z MMEF25-75) and peripheral blood monocyte levels in OW/OB patients, but not in normal weight patients. At the same time, a statistically significant inverse relationship was found between peripheral blood eosinophil levels and these spirometric parameters in normal weight patients, but not in OW/OB patients. The effect of sex on these associations was not statistically significant in our study. This study was the first to be conducted in a cohort of children and adolescents with BA.

T2 cell-mediated eosinophilic airway inflammation is currently considered to be the main mechanism of BA formation in normal weight children and adolescents [27], and total serum IgE levels and absolute eosinophil counts in peripheral blood act as systemic biomarkers of this type of inflammation. However, under the influence of OW/OB, T2-dependent inflammation may be modified, which may be associated with a decrease in the levels of these biomarkers [28].

In our sample of patients, average total IgE levels were slightly higher in normal weight children compared with the OW/OB group, but the differences were not statistically significant (*p* = 0.286). At the same time, the number of eosinophils was statistically significantly higher in normal weight patients compared to OW/OB patients (*p* = 0.039). The results of our study are consistent with those of other authors who have shown that OB may be associated with a noneosinophilic type of inflammation in patients with BA. According to the results of a meta-analysis by Nyambuya et al., systemic immune responses in patients with BA and OB are less associated with atopy [28]. The systemic inflammation in these patients differs from the classical atopic inflammation in normal weight asthmatics.

In our study, total leukocytes and also monocytes were statistically significantly higher in BA patients combined with OW/OB than in BA patients with normal body weight (all *p* < 0.001). We did not find any data in the literature on the number of leukocytes and monocytes in the peripheral blood of patients with different BMI in children and adolescents with asthma. In our study, z BMI in children and adolescents with BA was shown to have a statistically significant direct relationship with leukocyte and monocyte counts, but the opposite was true for peripheral blood eosinophil counts. Apparently, an increase in leukocytes and monocytes levels in the peripheral blood of BA patients with OW or OB is a universal sign of low-intensity systemic inflammation initiated by excess adipose tissue [29].

Our results are consistent with those of other studies. Yoshimura et al. showed that the total number of leukocytes and monocytes was significantly higher in OB adults compared to normal weight adults [17]. According to Jeong et al., a higher BMI is associated with increased leukocyte levels in peripheral blood [30]. By Jamshidi and Seif, peripheral blood leukocytosis is a characteristic feature of inflammation in OB and is caused by a chronic, sluggish inflammatory state [29]. Thus, our data are consistent with the notion that the processes of eosinophilic inflammation associated with atopy and the T2-dependent immune response in patients with BA and OB may be modified under the influence of low-intensity inflammation induced by adipose tissue and largely associated with monocytes.

It is unclear to what extent this alteration in the systemic inflammatory response may influence the airway inflammation that underlies the pathogenesis of asthma. Our data show a statistically significant inverse relationship between the absolute number of circulating monocytes and the decrease in z FEV1/FVC in patients with BA combined with OW/OB, but not in normal weight patients. We found only one study showing an inverse correlation between the total number of monocytes in the peripheral blood and FEV1/FVC in patients with severe asthma [24].

Airflow limitation may be associated with the development of meta-inflammation characteristic of OB [31]. This inflammation is realised with the involvement of a spectrum of cellular, humoral, metabolic, endocrine and neurogenic factors. One of the cellular effectors of this inflammation is monocytes, which may explain the inverse correlation between monocyte count and bronchial patency.

At the same time, in BA patients with normal body weight, these spirometric parameters had a statistically significant inverse relationship with the absolute number of eosinophils in the peripheral blood, which was *R* = −0.20, *p* = 0.021 for z FEV1/FVC. This relationship was not observed in patients with BA and OW/OB (all *p* > 0.05).

Our results are consistent with those of Hancox et al. who found a negative relationship between peripheral blood eosinophil count and spirometric parameters reflecting bronchial patency, including the FEV1/FVC ratio [5]. However, the influence of BW on this relationship was not considered in this work. In our study, there were no fundamental sex differences in the effect of BMI on monocyte and eosinophil levels and their relationship with spirometric parameters in children and adolescents with asthma. However, we did not study the sexual development of the patients on the Tanner scale.

The relationship between asthma and OB is complex and not fully understood. There is increasing evidence that OB is associated with low-intensity systemic inflammation, which may interact with and modify allergic inflammation in asthma [6]. And this may affect the efficacy of basic anti-inflammatory therapy with glucocorticoids in OW/OB BA patients [32].

### 4.1. Limitations

There are some limitations to this study. This was a single-centre study, and participants were not randomly recruited. The cross-sectional design does not allow us to draw conclusions about causality. In addition, normal-weight children/adolescents without BA and OW children/adolescents without BA could be studied as control groups. All participants were patients with mild to moderate asthma who had not received systemic steroid therapy for at least the previous 4 weeks.

A primary consequence of using a single-centre design is the potential lack of diversity in the study sample. Individuals recruited from a single-centre may not accurately represent a wider population, resulting in limited the generalisability of the findings to other conditions or populations. In addition, studies with a single-centre design may have less external validity than those conducted in a multicentre setting. Undoubtedly, replicating the findings of a study conducted at a single centre in diverse settings or with varied populations is crucial to validate the results and enhance their generalisability. Therefore, our future plans include a multicentre study to extend the applicability of the results to a wider range of geographical or demographic groups within the population.

Limitations of the study also include the fact that activity was only assessed through a survey, without the use of validated scales. In the future, we also plan to perform spirometry and UAC assessment in OW and obese children without BA.

## 5. Conclusion

Our results may confirm the hypothesis of a modification of eosinophilic inflammation in BA patients under the influence of OW/OB. The results obtained add to the information on the relationship between BMI and peripheral blood cells in BA patients. Thus, our study may contribute to a better understanding of BA phenotype in OB and point in the direction of further research, including anti-inflammatory therapy to identify the targets most significant in the pathogenesis of this variant. Another point of effort will be on body weight control in BA patients.

## Figures and Tables

**Table 1:** Patient clinical characteristics and spirometric parameters.

Parameters	Total (*N* = 212)	Normal weight (*N* = 135)	Overweight/obesity (*N* = 77)	*p* value
Age, years				
Total, *n* = 212	12.0 (9.0; 14.0)	11.0 (9.0; 15.0)	12.0 (10.0; 14.0)	0.850
Boys, *n* = 153	12.0 (10.0; 14.0)	11.0 (9.0; 14.0)	12.0 (10.0; 13.0)	*p* = 0.270
Girls, *n* = 59	12.0 (9.0; 15.0)	12.0 (9.0; 15.0)	11.0 (9.0; 14.0)	
z height	0.62 (−0.01; 1.29)	0.42 (−0.17; 1.06)	0.92 (0.50; 1.77)	**p** < 0.001
z weight	0.57 (0.22; 1.26)	0.44 (−0.31; 1.22)	0.84 (0.51; 1.31)	**p** < 0.001
z BMI	0.57 (−0.22; 1.31)	−0.02 (−0.43; 0.5)	1.54 (1.29; 1.95)	**p** < 0.001
z FVC	1.24 ± 1.15	1.17 ± 1.14	1.29 ± 1.14	0.080
z FEV_1_/FVC	−1.37 ± 1.15	−1.21 ± 1.22	−1.66 ± 0.95	**0.006**
z MMEF_25-75_	−1.18 ± 1.24	−1.09 ± 1.30	−1.29 ± 1.12	0.150

*Note:* z height, z weight, z BMI: z-score height, z weight, z BMI (body mass index); z FVC: z-score forced vital capacity; z FEV_1_/FVC: z-score FEV_1_ (forced expiratory volume in one second)/FVC ratio; z MMEF_25-75_: z-score forced expiratory flow between 25% and 75% of vital capacity. Values corresponding to the significance level *p* < 0.05 are marked in bold.

**Table 2:** Peripheral blood counts of study participants.

Parameters	Total (*N* = 212)	Normal weight (*N* = 135)	Overweight/obesity (*N* = 77)	*p* value
Erythrocytes (10^∗12^/L)	4.99 ± 0.44	4.96 ± 0.44	5.11 ± 0.43	0.085
Haemoglobin (g/L)	140.0 (132.0; 149.0)	139.0 (132.0; 148.0)	142.0 (132.0; 153.0)	0.099
Leukocytes (10^∗9^/L)	6.76 (6.07; 7.95)	6.61 (5.54; 7.49)	7.16 (6.53; 8.77)	**p** < 0.001
Monocytes (10^∗9^/L)	0.57 ± 0.17	0.54 ± 0.15	0.62 ± 0.19	**< 0.001**
Eosinophils (10^∗9^/L)	0.28 (0.13; 0.52)	0.3 (0.14; 0.56)	0.22 (0.12; 0.42)	**0.039**
Ig E (ME/L)	177.33 ± 228.43	191.77 ± 255.61	153.18 ± 170.49	0.286

*Note:* Values corresponding to the significance level *p* < 0.05 are marked in bold.

## Data Availability

The data that support the findings of this study are available on request from the corresponding author. The data are not publicly available due to privacy or ethical restrictions.
